# Further Increasing the Accuracy of Characterization of a Thin Dielectric or Semiconductor Film on a Substrate from Its Interference Transmittance Spectrum

**DOI:** 10.3390/ma14164681

**Published:** 2021-08-19

**Authors:** Dorian Minkov, Emilio Marquez, George Angelov, Gavril Gavrilov, Susana Ruano, Elias Saugar

**Affiliations:** 1 College of Energy and Electronics, Technical University of Sofia, 2140 Botevgrad, Bulgaria; g.gavrilov@tu-sofia.bg; 2 Department of Condensed-Matter Physics, Faculty of Science, University of Cadiz, 11510 Puerto Real, Spain; emilio.marquez@uca.es; 3 Department of Microelectronics, Technical University of Sofia, 1000 Sofia, Bulgaria; angelov@ecad.tu-sofia.bg; 4 Photovoltaic Solar Energy Unit, Energy Department, CIEMAT, Avenida Complutense 40, 28040 Madrid, Spain; susanamaria.fernandez@ciemat.es (S.R.); elias.saugar@csic.es (E.S.)

**Keywords:** increased characterization accuracy, thin film, envelope method, dielectric film, semiconductor film, transmittance spectrum

## Abstract

Three means are investigated for further increasing the accuracy of the characterization of a thin film on a substrate, from the transmittance spectrum *T*(*λ*) of the specimen, based on the envelope method. Firstly, it is demonstrated that the accuracy of characterization, of the average film thickness $\bar{d}$ and the thickness non-uniformity ∆d over the illuminated area, increases, employing a simple dual transformation utilizing the product *T*(*λ*)*x*_s_(*λ*), where *T*_sm_(*λ*) is the smoothed spectrum of *T*(*λ*) and *x*_s_(*λ*) is the substrate absorbance. Secondly, an approach is proposed for selecting an interval of wavelengths, so that using envelope points only from this interval provides the most accurate characterization of $\bar{d}$ and ∆d, as this approach is applicable no matter whether the substrate is transparent or non-transparent. Thirdly, the refractive index *n*(*λ*) and the extinction coefficient *k*(*λ*) are computed, employing curve fitting by polynomials of the optimized degree of 1/λ, instead of by previously used either polynomial of the optimized degree of λ or a two-term exponential of λ. An algorithm is developed, applying these three means, and implemented, to characterize a-Si and As_98_Te_2_ thin films. Record high accuracy within 0.1% is achieved in the computation of $\bar{d}$ and *n*(*λ*) of these films.

## 1. Introduction

Optical dielectric and semiconductor thin films have numerous applications in research and development, in applied sciences and engineering [1,2,3]. For example, such films, with thicknesses of at least 300 nm, are used in solar cells [4,5], thin-film transistors [6,7], photonic circuits [8,9], holography [10,11], and thin-film batteries [12,13]. Since the optical properties of these kinds of films depend on their composition and technology of preparation, there is a quest for increasing the accuracy of the optical characterization of the films [14,15].

**Figure 1:**
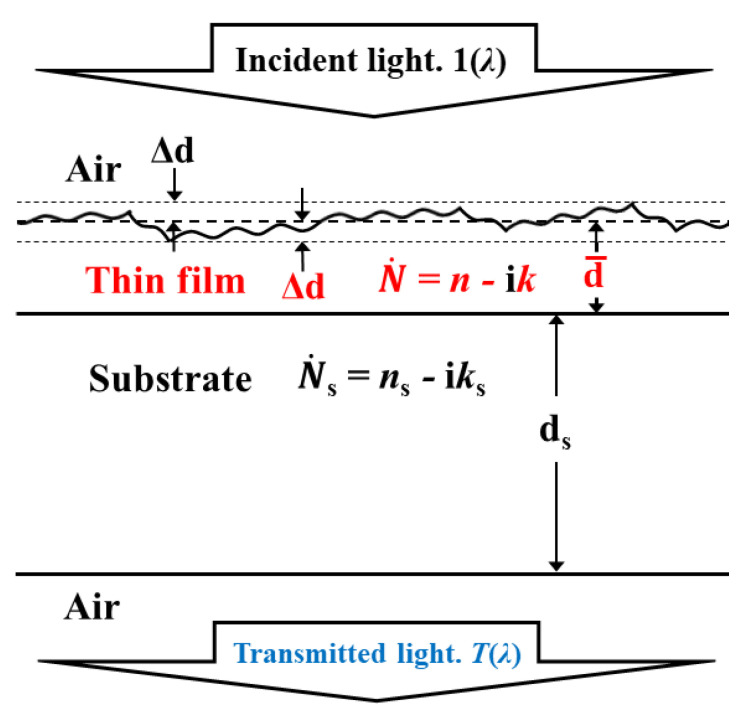
Sketch of transmittance *T*(*λ*) of light through a sample consisting of a thin film on a substrate. The thin film and its main optical characteristics are represented in red. ∆d is half of the difference between the maximum value and the minimum value of the film thickness *d* over the illuminated area on the free surface of the film.

The main approach for the optical characterization of a thin dielectric or semiconductor film is founded on the analysis of the transmittance spectrum *T*(*λ*) of a sample consisting of the film on a substrate, at normal light incidence to the film [16,17,18]. *T*(*λ*) is usually obtained by a spectrophotometer or a spectroscopic ellipsometer, operating, e.g., in the UV/VIS/NIR spectral region, as the illuminated area on the free surface of the film has a typical size between 0.5 mm and 10 mm [19,20,21]. The primary goal of such analysis of *T*(*λ*) is to determine the spectral dependencies of the refractive index *n*(*λ*) and the extinction coefficient *k*(*λ*), as well as the average thickness $\bar{d}$ of the film over the illuminated area [22,23]. In many cases, the film thickness is not constant along the surface, whereas *T*(*λ*) depends significantly on such variation in the film thickness [24,25]. This can be accounted for by including thickness non-uniformity ∆d = [max(*d*) − min(*d*)]/2 ≥ 0 in the formula for *T*(*λ*), where *d* is the film thickness at a particular point within the illuminated area [26,27]. It should be pointed out that the thickness non-uniformity ∆d of the film differs from its root mean square surface roughness *R*_q_ [28]. A sketch of light transmittance *T*(*λ*) through a thin film on a substrate, including the main optical characteristics of the film and the substrate, is shown in Figure 1. Notably, all the substrate characteristics are denoted by the subscript “s” throughout this paper.

Besides, *T*(*λ*) in the UV/VIS/NIR region of a sample, consisting of a thin dielectric or semiconductor film on a light transmitting substrate, usually contains interference patterns, due to the thin-film interference [29,30]. Furthermore, the above discussed film characteristics in the VIS/NIR spectral region can be computed based on the envelope method (EM), employing the upper envelope *T*_+_(*λ*) and the lower envelope *T*_−_(*λ*) of the smoothed spectrum *T*_sm_(*λ*) of *T*(*λ*) with interference pattern. Such *T*_sm_(*λ*) is tangent to and touches either of its envelopes *T*_+_(*λ*) or *T*_−_(*λ*) in several points, whose respective wavelengths *λ*_t_ are named tangency wavelengths. Importantly, EM does not use any dispersion model of *n*(*λ*) and/or *k*(*λ*), unlike the ellipsometric methods [31,32]; keeping in mind that such dispersion models are usually inaccurate for doped films [33,34], organic films [35,36], and mechanically stressed films [37].

With respect to the above, the founding paper about the EM for *T*(*λ*) of Swanepoel [38] is the most cited publication regarding the optical characterization of thin films, with over 4670 citations, according to Google Scholar [39,40]. Nevertheless, the EM for *T*(*λ*) of Swanepoel, from [38], does not consider the thickness non-uniformity ∆d of the film, neither the light absorption in the substrate. Improvements in the EM for *T*(*λ*) have accounted for ∆d [41] and for the light absorption in commonly used glass substrates [42]. However, in the EM for *T*(*λ*), from [41,42], three subjectively chosen parameters have been used, and the envelopes have not been corrected for the substrate absorption.

An EM for *T*(*λ*), providing and using optimized values of these three parameters, was presented in [43]. Moreover, a comparative study [44] showed that the EM for *T*(*λ*), from [43], furnishes the most accurate characterization of two a-Si films (with very different average thicknesses ($\bar{d}$) amongst four methods, selected as most likely to render accurate characterization of such films. Furthermore, corrections of the envelopes of *T*_sm_(*λ*) obtained as in the case of the transparent substrate, only at *λ*_t_ corresponding to notable substrate absorption, resulted in increasing the accuracy of the characterization of these films [45].

The formula for *T*_sm_(*λ*) at normal light incidence, in the most important case of *n*(*λ*) > *n*_s_(*λ*), is as follows [43,45]:

(1) $$T_{sm}{\left(\lambda \right)}_{}{=}_{}\frac{1}{\varphi_{2}-\varphi_{1}}\int_{\varphi_{1}}^{\varphi_{2}}dT_{u}{\left(\varphi \right)}_{}{=}_{}\frac{{\left(\tau_{a,f}\tau_{f,s}\tau_{s,a}\right)}^{2}x_{s}}{\varphi_{2}-\varphi_{1}}\int_{\varphi_{1}}^{\varphi_{2}}{\frac{x_{}d\varphi }{a_{1}-b_{1}cos\left(\varphi \right)+c_{1}sin\left(\varphi \right)}}_{},$$

where

$$\varphi {=}_{}4\pi nd/\lambda {,}_{}\varphi_{1}{=}_{}4\pi n(\bar{d}-\Delta d)/\lambda {,}_{}\varphi_{2}{=}_{}4\pi n(\bar{d}+\Delta d)/\lambda {,}_{}x{=}_{}exp(-4\pi kd/\lambda ){,}_{}$$

$$x_{s}{=}_{}exp(-4\pi k_{s}d_{s}/\lambda ){,}_{}a_{1}{=}_{}1-{\left(\rho_{a,f}\rho_{s,a}xx_{s}\right)}^{2}+\rho_{f,s}{}^{2}(\rho_{a,f}{}^{2}x^{2}-\rho_{s,a}{}^{2}x_{s}{}^{2}),$$

$$b_{1}=2\rho_{a,f}\rho_{f,s}\rho_{s,a}x_{}[\rho_{s,a}x_{s}{}^{2}cos\Delta_{2}-\rho_{s,a}{}^{-1}cos\Delta_{1}]{,}_{}c_{1}=2\rho_{a,f}\rho_{f,s}\rho_{s,a}x_{}[\rho_{s,a}x_{s}{}^{2}sin\Delta_{2}-\rho_{s,a}{}^{-1}sin\Delta_{1}],$$

$$\tau_{a,f}\tau_{f,s}\tau_{s,a}=\frac{8}{\sqrt{{\left(n+1\right)}^{2}+k^{2}}}\sqrt{\frac{n^{2}+k^{2}}{{\left(n+n_{s}\right)}^{2}+{\left(k+k_{s}\right)}^{2}}}\sqrt{\frac{n_{s}{}^{2}+k_{s}{}^{2}}{{\left(n_{s}+1\right)}^{2}+k_{s}{}^{2}},}$$

$$\rho_{a,f}=\sqrt{\frac{{\left(n-1\right)}^{2}+k^{2}}{{\left(n+1\right)}^{2}+k^{2}}},\rho_{f,s}=\sqrt{\frac{{\left(n-n_{s}\right)}^{2}+{\left(k-k_{s}\right)}^{2}}{{\left(n+n_{s}\right)}^{2}+{\left(k+k_{s}\right)}^{2}}},\rho_{s,a}=\sqrt{\frac{{\left(n_{s}-1\right)}^{2}+k_{s}{}^{2}}{{\left(n_{s}+1\right)}^{2}+k_{s}{}^{2}}},$$

$$\begin{array}{l} \Delta_{1}=tan^{-1}\left(\frac{2k}{n^{2}+k^{2}-1}\right)+\pi +tan^{-1}\left[\frac{2(kn_{s}-k_{s}n)}{n^{2}-n_{s}{}^{2}+k^{2}-k_{s}{}^{2}}\right],\\ \Delta_{2}=tan^{-1}\left(\frac{2k}{n^{2}+k^{2}-1}\right)+\pi -tan^{-1}\left[\frac{2(kn_{s}-k_{s}n)}{n^{2}-n_{s}{}^{2}+k^{2}-k_{s}{}^{2}}\right], \end{array}$$

where *T*_u_(*λ*) represents the transmittance of a film of the same material with uniform thickness *d* = $\bar{d}$, on the same substrate, and *x*(*λ*) is the absorbance of the film. It is assumed in Equation (1) that the film thickness *d* has a continuous uniform distribution in the interval [$\bar{d}$ − ∆d, $\bar{d}$ + ∆d] over the illuminated area, as the light passing through the film is considered to be coherent. The light passing through the substrate is regarded as non-coherent, thus quenching the light interference there, due to the significant thickness of the substrates used in practice [38,46].

The following accurate expressions for the upper envelope *T*_+_(*λ*) and lower envelope *T*_−_(*λ*) of *T*_sm_(*λ*), from Equation (1), have been presented in [47]:

(2) $$T_{\pm }(\lambda )=\frac{1}{\varphi_{2}{}_{{}_{\pm }}-\varphi_{1_{\pm }}}\int_{\varphi_{1_{\pm }}}^{\varphi_{2_{\pm }}}T_{u}(\varphi_{\pm })d\varphi_{\pm }=\frac{{\left(\tau_{a,f}\tau_{f,s}\tau_{s,a}\right)}^{2}x_{s}}{\varphi_{2}{}_{{}_{\pm }}-\varphi_{1_{\pm }}}\int_{\varphi_{1_{\pm }}}^{\varphi_{2_{\pm }}}{\frac{x_{}d\varphi_{\pm }}{a_{1}-b_{1}cos\left(\varphi_{\pm }\right)+c_{1}sin\left(\varphi_{\pm }\right)}}_{},$$

where

$$\begin{array}{l} \varphi_{+}=4\pi n(d-\bar{d})/\lambda {,}_{}\varphi_{1_{+}}=-4\pi n\Delta d/\lambda {,}_{}\varphi_{2_{+}}{=}_{}4\pi n\Delta d/\lambda ,\\ \varphi_{-}=\varphi_{+}+\pi {,}_{}\varphi_{1_{-}}=\varphi_{1_{+}}+\pi {,}_{}\varphi_{2-}=\varphi_{2_{+}}+\pi_{}, \end{array}$$

as “+” from the “±” signs refers to *T*_+_(*λ*), and “−” to *T*_−_(*λ*).

It is observed, from Equations (1) and (2), that *T*(*λ*), *T*_+_(*λ*), and *T*_−_(*λ*) are proportional to the first degree of the substrate absorbance *x*_s_(*λ*), whereas the dependencies of *x*_s_(*λ*) on *a*_1_, *b*_1_, and *c*_1_ can be neglected, since the interference in *T*_sm_(*λ*) indicates that *n*_s_(*λ*) >> *k*_s_(*λ*). Based on this, a dual transformation, consisting of forward and reverse transformations, was proposed in [47], for computing the envelopes *T*_+_(*λ*) and *T*_−_(*λ*) of *T*_sm_(*λ*), for a sample with a non-transparent substrate. The forward transformation includes the calculation of *T*(*λ*)′ *= T*(*λ*)/*x*_s_(*λ*) and its smoothing providing *T*_sm_(*λ*)′, which represents an approximation of the transmittance of the specimen whose substrate is replaced by a transparent one. The envelopes *T*_+_(*λ*)′ and *T*_−_(*λ*)′ of *T*_sm_(*λ*)′ are computed by substituting *x*_s_(*λ*)′ = *x*_s_(*λ*)/*x*_s_(*λ*) = 1 and using “boundary points”, “additional points”, “supplementary points”, and iterations, as in [35]. The tangency wavelengths *λ*_t_′ of *T*_sm_(*λ*)’, and its envelopes *T*_+_(*λ*)′ and *T*_−_(*λ*)’, are determined next. In the reverse transformation, *T*_sm_(*λ*) *= T*_sm_(*λ*)’*x*_s_(*λ*), and its envelopes *T*_+_(*λ*) = *T*_+_(*λ*)’*x*_s_(*λ*) and *T*_−_(*λ*) = *T*_−_(*λ*)’*x*_s_(*λ*) are calculated. Importantly, the tangency wavelengths *λ*_t_ of *T*_sm_(*λ*) are identical with the known *λ*_t_’, because *T*_sm_, *T*_+_, and *T*_−_ are derived by multiplying their respective *T*_sm_’, *T*_+_′ ≥ *T*_sm_′ and *T*_−_′ ≤ *T*_sm_′ by the same *x*_s_ > 0, for every *λ*.

The advantage of the above procedure for the computation of the envelopes of *T*(*λ*), is that it is based on using the envelopes of *T*_sm_(*λ*)′ (for a sample with transparent substrate, i.e., with *x*_s_(*λ*)′ = 1), which is not distorted by commonly observed kinks in *x*_s_(*λ*). This should increase the accuracy of *T*_+_(*λ*), *T*_−_(*λ*), and *λ*_t_; compared with correcting *T*_+_(*λ*) and *T*_−_(*λ*), obtained as for the transparent substrate, only at *λ*_t_ from the region with distinct *x*_s_(*λ*) < 1, as in [45].

Also, in any EM for *T*(*λ*), for the characterization of a thin film with *n*(*λ*) > *n*_s_(*λ*), at normal light incidence, the following interference condition is used:

(3) $$2_{}n{\left(\lambda_{t}\right)}_{}\bar{d}=m_{\ell }(\lambda_{t})\lambda_{t}{\left(\ell \right)}_{}{}_{}\{\begin{array}{c} m_{\ell }{}_{}\geq_{}1_{}{-}_{}integer\;for\;all\;tangency\;wavelengths\;\lambda_{t}{\left(\ell \right)}_{}from\;the\;envelope\;T_{+}(\lambda )\\ m_{\ell }{}_{}\geq_{}1/2_{}{-}_{}half-integer\;for\;all\;tangency\;wavelengths\;\lambda_{t}\left(\ell \right)\;from\;the\;envelope\;T_{-}(\lambda ), \end{array}$$

where *ℓ* = 1, 2, … *ℓ* _M_ is the number of the tangency wavelength *λ*_t_, counted from the longer wavelengths end of *T*(*λ*), and *m_ℓ_* (*λ*_t_) is the interference order.

Furthermore, all versions of the envelope method for thin-film characterization are executed in two stages, as in the first stage, $\bar{d}$, ∆d (if employed), and the lowest interference order *m*_1_[max(*λ*_t_)] are computed; and in the second stage, *n*(*λ*) and *k*(*λ*) are computed. Taking into account that such EM characterizations have been commonly performed using consecutive tangency wavelengths *λ*_t_(*ℓ* ), including the longest *λ*_t_(*ℓ* =1), the following error metric was proposed in [47], for estimating the accuracy of the first stage of the characterization:

(4) $$\delta d/{\bar{d}}_{}(1)=\left(1/{\bar{d}}_{1}\right)\sqrt{\frac{\sum_{{}_{{}_{}}\ell =1_{{}^{}}}^{{}_{{}_{}}\ell_{0_{}}}{\left\{{\bar{d}}_{1}{}_{}{-}_{}{\bar{d}}_{c}\left[\lambda_{t}(\ell )\right]\right\}}^{2}}{\ell_{0_{}}}}\geq 0_{},$$

where $\bar{d}$_c_[*λ*_t_(*ℓ*)] is the average film thickness calculated from Equation (3), for the tangency wavelength *λ*_t_(*ℓ*), *ℓ*_0_ is the number of all the tangency wavelengths participating in the first stage of the characterization, and $\bar{d}$_1_ is the mean value of $\bar{d}$_c_[*λ*_t_(*ℓ*)]. Since the smaller variation of $\bar{d}$_c_[*λ*_t_(*ℓ*)] over *ℓ* = [1, 2, … *ℓ* _0_] points to more accurate computation of $\bar{d}$ and ∆d of the film [38,43], the most accurate first-stage EM characterization of the film corresponds to the smallest δd/$\bar{d}$ in Equation (4). In this regard, EM characterization of thin a-As_x_Te_100−x_ films, using the dual transformation for computing *T*_+_(*λ*) and *T*_−_(*λ*), resulted in much smaller δd/$\bar{d}$ compared with the EM characterization of thin a-Si films using envelopes that are corrected only at *λ*_t_ from the region with distinct *x*_s_ < 1.

The second stage of EM characterization starts with the calculation of approximations *n*_0_(*λ*_t_) of the refractive index of the film, from Equation (3), followed by computation of the refractive index *n*(*λ*) of the film by curve fitting to *n*_0_(*λ*_t_). The extinction coefficient *k*(*λ*) of the film is computed last, by solving one equation for every *λ*; usually from *T*_+_(*λ*) [38,41,42], or from the following [47]:

(5) $$T_{i}(\lambda )={\sqrt{{}_{}T_{+}{\left(\lambda \right)}_{}T_{-}(\lambda )}}_{}$$

In [43], the extinction coefficient of the film was computed from *T*_sm_(*λ*), as *k*(*λ*) = *k*_0_(*λ*) + Δ*k*(*λ*), where *k*_0_(*λ*) is determined by curve fitting to the approximations *k*_a_(*λ*_t_) that were obtained by solving Equation (1), and the correction Δ*k*(*λ*) is a half-sum of the two envelopes of the difference *k*_a_(*λ*) − *k*_0_(*λ*). Notably, the curve-fitting functions that were employed in [45], for the computation of *n*(*λ*) and *k*_0_(*λ*), were either polynomial of the optimized degree of *λ* or a two-term exponential of *λ.*

The accuracy of a given thin-film characterization can be assessed based on the computation of a reconstructed transmittance spectrum *T*_r_(*λ*), by replacing $\bar{d}$, ∆d, *n*(*λ*), and *k*(*λ*) in the right side of Equation (1), and comparison of *T*_r_(*λ*) with *T*(*λ*). A measure of the closeness of *T*_r_(*λ*) to *T*(*λ*) is the figure of merit [45], as follows:

(6) $$FOM{=}_{}{\sqrt{\frac{\sum_{j=1}^{N_{j}}{}_{}{\left\{T{\left[\lambda (j)\right]}_{}{-}_{}T_{r}\left[\lambda (j)\right]\right\}}^{2}}{N_{j}}}}_{}\geq_{}0$$

with summation over all *λ*⊂[min(*λ*_t_), *λ*_t_(*ℓ* =1)], whereas a smaller *FOM* corresponds to more accurate film characterization.

From the above comments three issues are identified, regarding further increasing the accuracy of the characterization of a thin dielectric or semiconductor film on a substrate, based on the EM for *T*(*λ*). These issues are presented below, in the order of their appearance in the algorithm of the EM for *T*(*λ*). The first issue is to study which envelopes, *T*_+_(*λ*) and *T*_−_(*λ*), should be chosen for the non-transparent substrate; those computed as for a transparent substrate and corrected only at *λ*_t_, with distinct *x*_s_(*λ*_t_) < 1, or those obtained by a dual transformation for all *λ*. The second issue is to establish whether an interval *ℓ* = [*ℓ* _1_, *ℓ* _2_] (representing only the used *λ*_t_), over which the first stage of the characterization is performed most accurately, can be selected, compared with the commonly used interval *l* = [1, *ℓ* _0_]. The third issue is to explore the concept of using a regression of *n*(*λ*) and *k*_0_(*λ*), by a polynomial of an optimized degree of 1/*λ* (consistent with the Cauchy’s dispersion formula [48,49]), instead of by either the polynomial of an optimized degree of *λ* or a two-term exponential of *λ* [45,47].

In this paper, the above three issues are investigated by performing characterizations of relatively thick a-Si and a-As_98_Te_2_ thin films that are deposited on non-transparent glass substrates, based on the EM for *T*(*λ*). An algorithm is proposed for further increasing the accuracy of the characterization of thin dielectric or semiconductor film on a substrate, employing such EM for *T*(*λ*), and the characterization accuracy of the studied films is increased by using this algorithm.

## 2. Materials and Methods

### 2.1. Preparation of the Specimens and Measuring the Transmittance Spectra T(λ)

The a-Si film has been deposited on 3.28-mm-thick Borofloat33 glass substrate by RF magnetron sputtering using RF power of 525 W, Ar gas with pressure of 0.13 Pa, and target-to-substrate distance of 6.1 cm, as described in [45]. The transmittance spectrum *T*(*λ*) of this specimen has been measured using a Perkin-Elmer Lambda 1050 UV/VIS/NIR spectrophotometer, with slit-width set at 2 nm, data collection interval of 1 nm, normal light incidence to the film, and illuminated area of 10 mm × 3 mm [50].

The As_98_Te_2_ film has been prepared by plasma-enhanced chemical vapor deposition for fifteen minutes on 1-mm-thick standard microscope slide glass substrate of Levenhuk, as described in [51]. *T*(*λ*) of this specimen has been measured using a Cary 5000 double-beam spectrophotometer of Agilent, with slit-width of 3.44 nm, data collection interval of 1 nm, normal light incidence to the film, and a circular illuminated area with 1 mm diameter [52].

### 2.2. Details Regarding the Performed Accurate Thin Film Characterizations by EM for T(λ)

For both studied specimens, the refractive index of the substrate *n*_s_(*λ*) and its extinction coefficient *k*_s_(*λ*) are calculated by solving the system of two well-known equations for the transmittance *T*_s_(*λ*) and the reflectance *R*_s_(*λ*) of the bare substrate, which have also been measured [43,47].

It is pointed out that the thin-film characterizations reported in this study are founded on the EM for *T*(*λ*) from [43,45], as the dual transformation for computing *T*_+_(*λ*) and *T*_−_(*λ*) is adopted from [47]. Regarding the proposed selection of an interval *ℓ* = [*ℓ* _1_, *ℓ* _2_], over which the first stage of the characterization is performed most accurately, it is realized by including a program cycle increasing the integer *ℓ* _1e_ (representing *ℓ* _1_), starting from *ℓ* _1e_ = 1. Moreover, one of the three optimized parameters determined by the EM for *T*(*λ*) from [43] is the number of the tangency wavelengths *λ*_t_(*ℓ*), participating in the first stage of the characterization, denoted as *ℓ* _0_ in Equation (4). Based on the above and Equation (4), the error metric employed for computing $\bar{d}$, ∆d and *m*_1_[max(*λ*_t_)] at the end of the first stage of the characterization is as follows:

(7) $$\delta d/{\bar{d}}_{}\left(\ell_{1e}\right)=\left(1/{\bar{d}}_{e}\right)\sqrt{\frac{\sum_{{}_{{}_{}}\ell =\ell_{1e}}^{{}_{{}_{}}\ell_{2e_{}}}{\left\{{\bar{d}}_{e}{}_{}{-}_{}{\bar{d}}_{ce}\left[\lambda_{t}(\ell )\right]\right\}}^{2}}{\ell_{2e_{}}-\ell_{1e_{}}+1}}\geq 0$$

where *ℓ* _2e_ is automatically computed by the EM for *T*(*λ*) from [33]. Correspondingly, *ℓ* _1_ = *ℓ* _1e_ for which δd/$\bar{d}$(*ℓ* _1e_) has a minimum value and *ℓ* _2_ is its respective *ℓ* _2e_, which completes the selection of the interval *ℓ* = [*ℓ* _1_,*ℓ*_2_].

Besides the accurate computation of *n*(*λ*) and *k*(*λ*) in the second stage of the characterization, their approximations can also be computed there by solving the system of two equations for *T*_+_(*λ*) and *T*_−_(*λ*), the computed result being denoted as (*n*, *k*) = f(*T*_+_, *T*_−_).

### 2.3. Algorithm for Accurate Thin-Film Characterization by EM for T(λ) Accounting for the Three Investigated Issues

**Figure 2:**
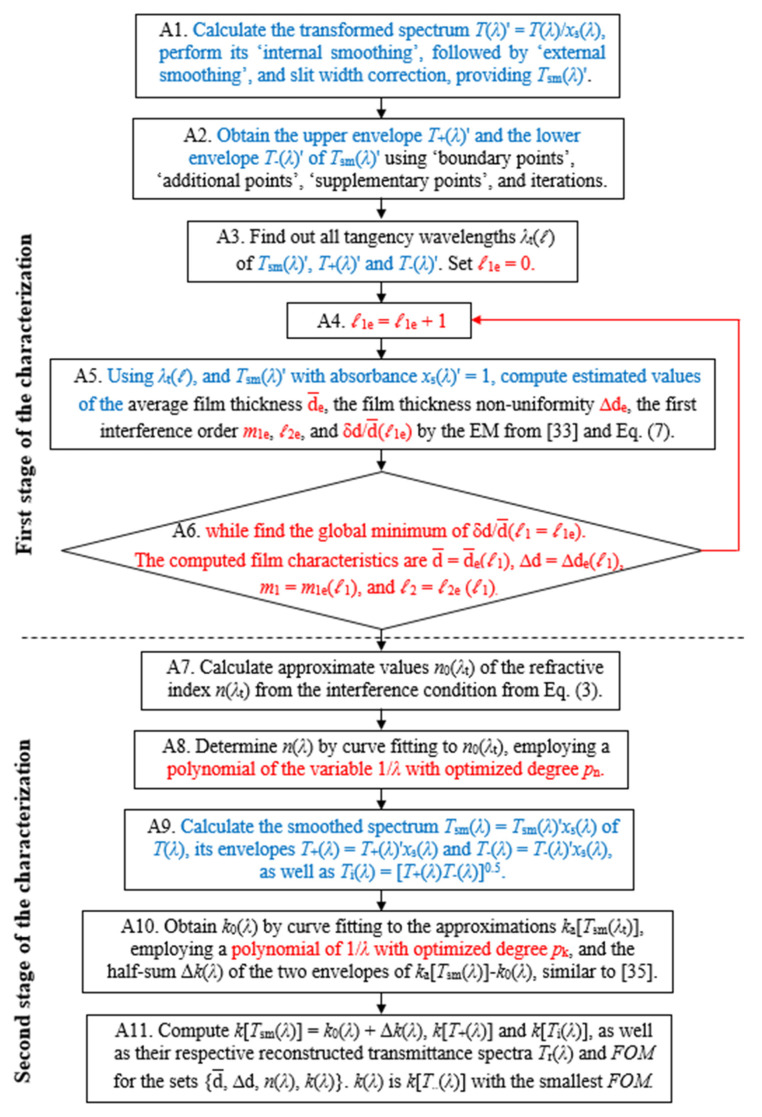
Algorithm for accurate thin-film characterization by EM for *T(λ)* accounting for the three investigated issues. The text in black corresponds to the algorithm from [45], the text in blue represents the dual transformation for computing the envelopes *T*_+_(*λ*) and *T*_−_(*λ*) of *T*_sm_(*λ*) from [47], as the text and arrow in red are related to the second and the third investigated issues.

An algorithm for accurate thin-film characterization accounting for the three investigated issues is developed, shown in Figure 2, and used in this study.

The above algorithm is founded on the algorithm of EM for *T*(*λ*) presented in [45], as the dual transformation for computing *T*_+_(*λ*) and *T*_−_(*λ*) is adopted from [47], and the second and third investigated issues represent novelties. In principle, the algorithm from Figure 2 optimizes and utilizes the three parameters *ℓ*_1_, *ℓ*_2_ and Δd, which provide best fit of the spectrum *T*_sm_(*λ*) (calculated from Equation (1)) to the experimental spectrum *T*(*λ*) in the interval [*λ*_t_(*ℓ*_1_), *λ*_t_(*ℓ*_2_)] from the spectral region of quasi-transparency and weak absorption of the film. To avoid using a local minimum of the error metric δd/$\bar{d}$ at step A6 of the algorithm (instead of the needed global minimum), the optimization of the above three parameters is achieved by a ‘direct search′ [53] employing all plausible values of these parameters. Notably, *T*_sm_(*λ*)’, featured at step A1, is obtained by external smoothing, similar to [45,47], which is beneficial for comparison of computed results from this paper with similar results from [45,47].

Furthermore, the estimated value $\bar{d}$_e_(*ℓ* _1e_) of the average film thickness (over the illuminated area) is calculated at step A5 by averaging the film thicknesses estimated for consecutive tangency wavelengths *λ*_t_, as described in [43]. Correspondingly, δd (formulated by Equation (7)), obtained at step A6, represents the absolute error in the computation of the average film thickness $\bar{d}$. Respectively, the global minimum of δd/$\bar{d}$(*ℓ* _1e_) (defined by Equation (7)), also computed at step A6, represents the relative error in the computation of the average film thickness $\bar{d}$. Moreover, since the refractive index *n*(*λ*) of the film is computed based on Equation (3), its left side being the product 2*n*(*λ*_t_)$\bar{d}$ and its right side being fixed for a given *λ*_t_, the global minimum of δd/$\bar{d}$(*ℓ* _1e_) also represents the relative error in the computation of *n*(*λ*).

With respect to the above paragraph, characterization of four types of model thin film on glass substrate samples was performed in [54], by the EM for *T*(*λ*) from [33] using the error metric δd/$\bar{d}$(*ℓ* _1e_ = 1). These four types of model films represent the four possible combinations of quasi-uniform or non-uniform dielectric or semiconductor film with or without a wide spectral region of quasi-transparency. The results from [54] have shown that this kind of characterization of such films should provide relative error │$\bar{d}$−$\bar{d}$_M_│/$\;\bar{d}$_M_ < 0.1% in the computation of the average film thickness (where $\bar{d}$_M_ is the known average film thickness of the model film), as well as δd/$\bar{d}$_M_ ≈ δd/$\bar{d}$ < 0.1%. However, this kind of characterization of the films studied here has led to minimum values of δd/$\bar{d}$ = 0.245% for the a-Si film, and δd/$\bar{d}$ = 0.133% for the a-As_98_Te_2_ film, as reported in [47], thus missing the above target of δd/$\bar{d}$ < 0.1%. Nevertheless, the above discussed data from [54] indicate that δd/$\bar{d}$ (computed by the algorithm from Figure 2) represents the relative error │$\bar{d}$−$\bar{d}$_true_│/$\;\bar{d}$_true_ (where $\bar{d}$_true_ is the true value of the average film thickness over the illuminated area) in the computation of the average film thickness, independent from the optical characteristics of the film.

### 2.4. Determination of Model Based Thin-Film Parameters Using Data from EM Characterization

A study of various amorphous semiconductors and glasses has shown that the dispersion of the refractive index *n*(*λ*) can be described by the Wemple-DiDomenico single-effective-oscillator model represented by the dependence, as follows:

(8) $$n(E)\;≃\;\sqrt{1+\frac{E_{0}E_{d}}{\left[E_{0}{}^{2}-E^{2}(\lambda )\right]}}$$

where *E*(eV) = 1239.8/*λ* (nm) is the photon energy, *E*_0_ > *E* is the oscillator energy and *E*_d_ is the oscillator strength [55]. On the other hand, a-Si and a-As are amorphous semiconductors, and in many cases binary arsenic chalcogenide films are also amorphous semiconductors [56,57]. Taking into account the above, in this paper is used Wemple-DiDomenico plot of {*n*(*E*(*λ*_t_))^2^ − 1}^−1^ versus *E*(*λ*_t_)^2^, which should be represented by a straight line corresponding to Equation (8), for the studied a-Si and a-As_98_Te_2_ thin films. Since the accuracy of Equation (8) might decrease with increasing *k*(*λ*), the parameters *E*_0_ and *E*_d_ are determined by a low-energy linear regression to the Wemple-DiDomenico plot, and the static refractive index of the film is expressed from Equation (8) as follows:

(9) $$n_{0}=n\left(E=0\right)=n\left(0\right)={\sqrt{1+E_{d}/E_{0}}}_{}.$$

Optical characteristics of other magnetron-sputtered a-Si thin films were studied in [50], depending on the pressure of the Ar gas, as the other technological parameters were identical to those for the a-Si thin film studied in this paper. The following empirical formula was obtained there:

(10) $$E_{g}\;\approx \;\left(8.3-n_{0}\right)/3.8_{}{}_{}(eV)$$

where *E*_g_ is the band gap of magnetron-sputtered a-Si thin film. Equation (10) can be used for estimation of the band gap of the studied a-Si thin film.

It is also known that for amorphous semiconductors the following Tauc equation is usually valid:

(11) $${\left(\alpha E\right)}^{1/2}≃B_{T}(E\;-\;E_{g})$$

for α(*E*) > 10^4^ cm^−1^, where α(λ) = 4π*k*(*λ*)/*λ* is the absorption coefficient of the film, and *B*_T_ is the Tauc slope [58]. Correspondingly, the band gap *Eg* can also be approximated employing Tauc plot of (α*E*)^1/2^ versus *E* and its high-energy linear regression with α(*E*) > 10^4^ cm^−1^, as *E*_g_ represents the photon energy at which the regression line crosses the horizontal axis [58]. In order to use Tauc plot, *n*(*E*) is usually calculated from Equation (8), by substituting *E*_0_ and *E*_d_ determined from the Wemple-DiDomenico plot (assuming validity of the single-effective-oscillator model), followed by computation of α(λ) from Equation (1).

Besides, disorder in the local structure of material leads to presence of localized electronic states in its band gap, as it is generally accepted that α(*E*) of amorphous materials tails off exponentially, in the following:

α(*E*) = α_0_exp(*E*/*E*_U_) (12)

in the interval α(*E*) ⊂ [10^3^, 10^4^] cm^−1^, where *E*_U_ is the Urbach energy and α_0_ is a pre-exponential factor [59,60]. Therefore, both *E*_U_ and α_0_ can be derived using Urbach energy plot of log_10_[α(cm^−1^)] versus *E* and its linear regression in the interval log_10_[α(cm^−1^)] ⊂ [3, 4]. Moreover, the photon energy corresponding to α(*E*) = 10^3^ cm^−1^ is denoted by *E*_03_, and the photon energy corresponding to α(*E*) = 10^4^ cm^−1^ is denoted by *E*_04_.

Furthermore, voids can occur in a-Si [61], and the voids volume fraction (compared to the entire volume of the material) has been approximated as follows:

(13) $$f_{void}\;≃\;\frac{\left[1+\;2n^{2}(0)\right]\left[n_{dense}^{2}(0)-n_{}^{2}(0)\right]}{3n_{}^{2}(0)\left[n_{dense}^{2}(0)-1\right]}$$

where *n*_dense_(0) is the static refractive index of a-Si without voids [50,62]. Besides, *E*_0_ = 2.873 eV and *E*_d_ = 36.404 eV have been reported for pure a-Si [63], and replacing these data in Equation (9) provides *n*_dense_(0) = 3.70. This result and Equation (13) permit calculation of the voids volume fraction for the studied a-Si thin film.

## 3. Results

### 3.1. Characterization of the a-Si Film

**Figure 3:**
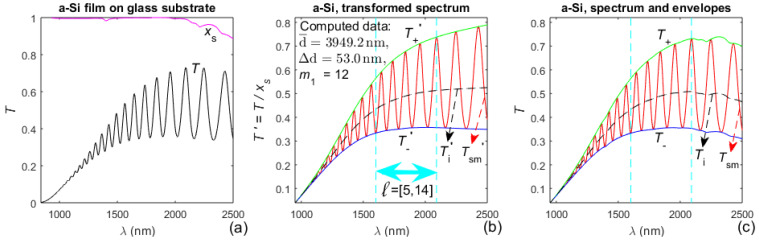
Transmittance-related spectra and results from the first-stage characterization of the a-Si film: (**a**) transmittance spectrum *T*(*λ*) of the specimen, and substrate absorbance *x*_s_(*λ*); (**b**) forward transformed spectra and results from the first stage characterization. The selected interval *ℓ* = [5,14] providing the most accurate first-stage characterization is marked by double arrow; (**c**) the reverse-transformed smoothed spectrum *T*_sm_(*λ*), its envelopes *T*_+_(*λ*) and *T*_−_(*λ*), and *T*_i_(*λ*) from Equation (5).

Illustrations about the first stage from the characterization of the a-Si film, by the algorithm from Figure 2, are presented in Figure 3.

The variation in the interval *ℓ* _e_ = [*ℓ* _1e_, *ℓ* _2e_] and its respective error metric δd/$\bar{d}$(*ℓ* _1e_) for the successively increasing *ℓ* _1e_ (in the process of selecting the interval *ℓ* = [*ℓ* _1_, *ℓ* _2_], providing most accurate first step characterization), are shown in Table 1 for the a-Si film.

**Figure 4:**
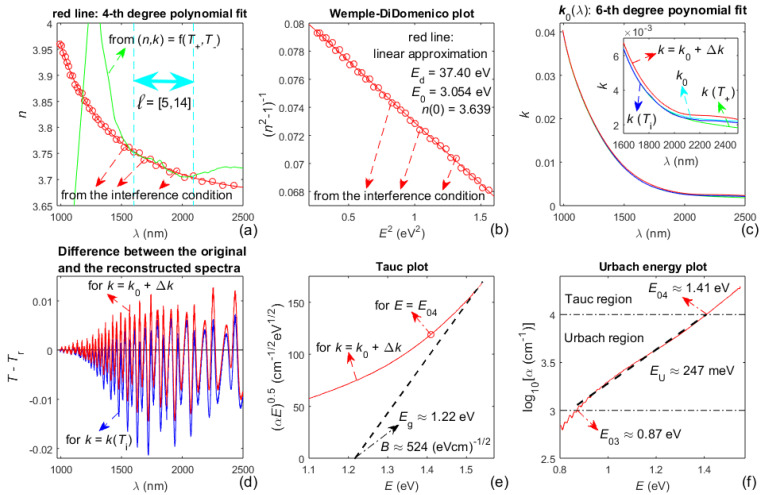
Illustrations regarding computed material characteristics of the a-Si film. (**a**) The refractive index *n*(*λ*) determined at steps A7 and A8 from the algorithm; (**b**) Wemple-DiDomenico plot; (**c**) data for the extinction coefficient *k*(*λ*) from steps A10 and A11; (**d**) differences *T*(*λ*)–*T*_r_(*λ*), from step A11, for *k* = *k*(*T*_i_) and *k* = *k*_0_ + Δ*k*; (**e**) Tauc plot; (**f**) Urbach energy plot.

Pictures concerning the rest of the characterization of the a-Si film, based on the algorithm from Figure 2, are displayed in Figure 4.

*FOM*, computed from Equation (6) at step A11 from the algorithm, is presented in Table 2, for several choices of the extinction coefficient *k*(λ) of the a-Si film.

### 3.2. Characterization of the a-As_98_Te_2_ Film

**Figure 5:**
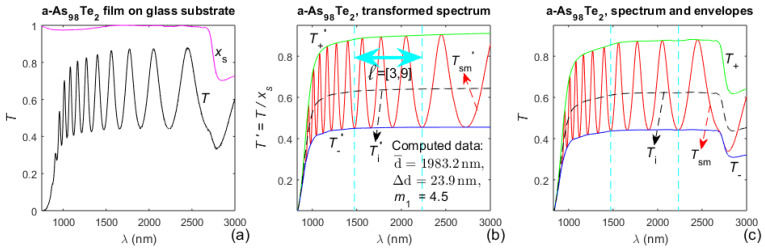
Transmittance-related spectra and results from the first-stage characterization of the a-As_98_Te_2_ film: (**a**) transmittance spectrum *T*(*λ*) of the specimen, and substrate absorbance *x*_s_(*λ*); (**b**) forward-transformed spectra and results from the first-stage characterization. The selected interval *ℓ* = [3,9] providing the most accurate first-stage characterization is marked by double arrow; (**c**) the reverse-transformed smoothed spectrum *T*_sm_(*λ*), its envelopes *T*_+_(*λ*) and *T*_−_(*λ*), and *T*_i_(*λ*) from Equation (5).

Illustrations about the first stage from the characterization of the a-As_98_Te_2_ film, by the algorithm from Figure 2, are presented in Figure 5.

The variation in the interval *ℓ* _e_ = [*ℓ* _1e_, *ℓ* _2e_] and its respective error metric δd/$\bar{d}$(*ℓ* _1e_), for the successively increasing *ℓ* _1e_ (in the process of selecting the interval *ℓ* = [*ℓ* _1_, *ℓ* _2_], providing most accurate first step characterization), are shown in Table 3 for the a-As_98_Te_2_ film.

**Figure 6:**
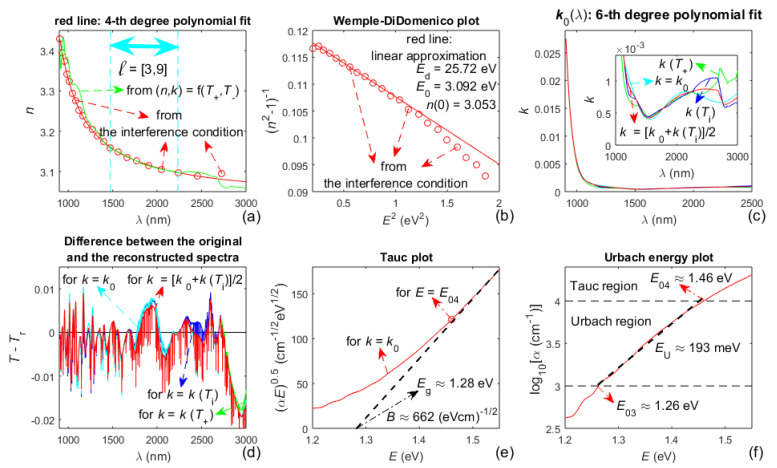
Illustrations regarding computed material characteristics of the As_98_Te_2_ film. (**a**) The refractive index *n*(*λ*) determined at steps A7 and A8 from the algorithm; (**b**) Wemple-DiDomenico plot; (**c**) data for the extinction coefficient *k*(*λ*) from steps A10 and A11; (**d**) differences *T*(*λ*)–*T*_r_(*λ*), from step A11, for *k* = *k*(*T*_i_), *k* = *k*_0_, *k* = *k*(*T*_+_) and *k* = [*k*_0_ + *k*(*T*_i_)]/2; (**e**) Tauc plot using *n*(*E*) calculated by a linear approximation of the higher-energy region from the Wemple-DiDomenico plot; (**f**) Urbach energy plot.

Pictures representing the rest of the characterization of the a-As_98_Te_2_ film, based on the algorithm from Figure 2, are displayed in Figure 6. *FOM*, computed from Equation (6) at step A11 from the algorithm, is presented in Table 4, for several choices of the extinction coefficient *k*(*λ*) of the a-As_98_Te_2_ film.

**Figure 7:**
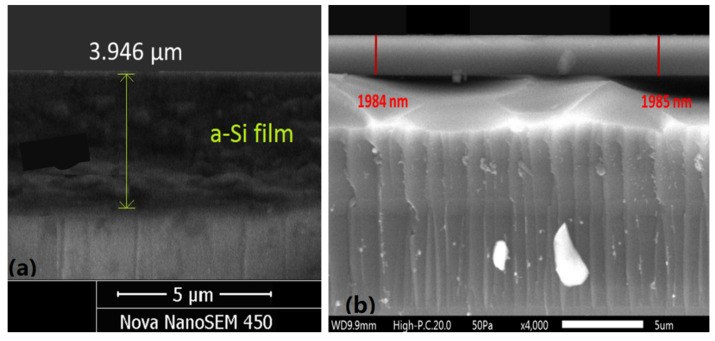
SEM images of cross-sections of the studied films. (**a**) for the a-Si film and (**b**) for the As_98_Te_2_ film.

SEM images of cross-sections of both studied films are shown in Figure 7.

## 4. Discussion

With respect to the presented dual transformation, it is observed from Figure 3b,c and Figure 5b,c that the forward transformation provides *T*_+_(*λ*)′ and *T*_−_(*λ*)′ without any kinks, unlike the envelopes *T*_+_(*λ*) and *T*_−_(*λ*) reproducing the kinks in *x*_s_(*λ*). This indicates that such dual transformation should furnish more accurate points *T*_+_(*λ*_t_) and *T*_−_(*λ*_t_) (needed for the first-stage characterization of the film), than using envelopes as the transparent substrate and correcting them only at *λ*_t_ with distinct *x*_s_(*λ*_t_) < 1, as in [45]. Indeed, according to the data from Table 1 about the a-Si film, the error metric δd/$\bar{d}$(*ℓ* _1e_ = 1) = 0.143% obtained in this study is significantly smaller than δd/$\bar{d}$(*ℓ* _1e_ = 1) = 0.245% from [45], which confirms the above presumption. Moreover, employing the dual transformation is significantly simpler than using envelopes as the transparent substrate and correcting them only at *λ*_t_ with distinct *x*_s_(*λ*_t_) < 1.

Regarding the selection of the interval *ℓ* = [*ℓ* _1_, *ℓ*_2_], over which the first-stage characterization is most accurate (i.e., the interval corresponding to the smallest δd/$\bar{d}$(*ℓ* _1e_), *ℓ* = [5,14] for the a-Si film and *ℓ* = [3,9] for the As_98_Te_2_ film, as observed from Table 1 and Table 3. Besides, a review of Figure 3a,b and Figure 5a,b shows that the selected interval spreads out over a spectral region with *x*_s_(*λ*)≃1, i.e., over a region where the substrate is quasi-transparent, for either of the studied films. This can be attributed to light scattering in the substrate, associated with the light absorption in the substrate, for wavelengths corresponding to *x*_s_(*λ*) < 1 [64]. Indeed, since light scattering in the substrate is not accounted for in the EM formulae, Equations (1) and (2) can predict somewhat larger values of the transmittance spectrum than the experimentally measured *T*(*λ*) in the region of distinct *x*_s_(*λ*) < 1. To mitigate this effect, the EM for *T*(*λ*) computes a visibly smaller extinction coefficient *k*(*λ*) of the film in the region of the smallest *x*_s_(*λ*) < 1; notably, such result is observed in the longest wavelength parts of Figure 4c, and especially of Figure 6c (since *x*_s_(*λ*) ≪ 1 for *λ* > 2700 nm). The above arguments indicate that the selection and employment of an interval *ℓ* = [*ℓ* _1_, *ℓ* _2_], over which the first-stage characterization is most accurate, is especially beneficial for the characterization of a film on a non-transparent substrate. However, since the envelopes *T*_+_(*λ*) and *T*_−_(*λ*) are less accurate in the long wavelength part of *T*(*λ*), e.g., due to luck of their precise boundary points there, use of such interval *ℓ* = [*ℓ* _1_, *ℓ* _2_] is expected to also be favorable for the characterization of a thin film on a transparent substrate.

Based on the above, the employment of both the dual transformation (for non-transparent substrate) and the selected interval *ℓ* = [*ℓ* _1_, *ℓ* _2_], corresponding to the smallest δd/$\bar{d}$(*ℓ* _1e_), should provide the most accurate thickness characteristics $\bar{d}$ and ∆d of the film.

However, the refractive index from (*n*,*k*) = f(*T*_+_, *T*_−_), computed by solving the equations for *T*_+_(*λ*) and *T*_−_(*λ*), is apparently inaccurate (compared to *n*(*λ*), obtained based on the interference condition), as observed from Figure 4a and Figure 6a. This is mainly due to the imperfection of the envelopes of *T*(*λ*), and indicates that *n*(*λ*) and *k*(*λ*) should not be computed only from Equation (2).

The concept of using a regression of *n*(*λ*) and *k*_0_(*λ*), by a polynomial of the optimized degree of 1/*λ*, has two advantages. First, it is consistent with the fact that the vast majority of dielectrics and semiconductors exhibit normal dispersion in the employed UV/VIS/NIR spectral region [19,21], as well as with its representation by the Cauchy’s dispersion formula [48,49]. Second, it eliminates the inconvenience of choosing a regression between a polynomial of the optimized degree of λ and a two-term exponential of λ, as in [45].

The Wemple-DiDomenico plot from Figure 4b illustrates that the dispersion of the refractive index *n*(*λ*) of the a-Si film is represented well by the single-effective-oscillator model and Equation (8). Besides, the static refractive index *n*_0_ = 3.639 of the a-Si film (sputtered using Ar gas with a pressure of 0.13 Pa), calculated from Equation (9), matches *n*_0_ ≈ 3.60 for similar films from [50]; inasmuch as *n*_0_ = 3.639 is quite close to *n*_dense_(0) = 3.70 for pure a-Si [63]. Moreover, replacing these values of *n*_0_ and *n*_dense_(0) in Equation (13) provides a voids volume fraction $f_{void}\;≃\;0$. The above facts indicate that the a-Si film studied here is quite dense and almost without voids.

The band gap *E*_g_ of the a-Si film is estimated by substituting *n*_0_ = 3.639 in Equation (10), which leads to *E*_g_ ≈ 1.20 eV, and *E*_g_ ≈ 1.22 eV is obtained by the Tauc plot from Figure 4e; whereas these data match *E*_g_ ≈ 1.23 eV for similar films from [50]. Furthermore, the Urbach energy *E*_U_ ≈ 247 meV, derived based on the Urbach energy plot from Figure 4f, is similar to *E*_U_ ≈ 266 meV for the respective films from [50]. Notably, it is observed from Table 2 that the minimum *FOM* achieved in this study is smaller than its respective value (for the same film and *k* = *k*_0_ + Δ*k*) from [45], which implies that the characterization of the a-Si film presented here is more accurate than that from [45].

Concerning the second stage from the characterization of the a-As_98_Te_2_ film, the Wemple-DiDomenico plot from Figure 6b shows that the single-effective-oscillator model does not represent *n*(*λ*) well over all the tangency wavelengths *λ*_t_ of *T*(*λ*). Correspondingly, in the Tauc plot from Figure 6e, *n*(*E*) is used, calculated by a linear approximation of the higher photon energy region from the Wemple-DiDomenico plot. Regarding Table 4, since relatively smaller *FOM* are obtained in this study, for *k* = *k*_0_ and *k*(*T***_i_**), as well as that these two quantities are derived independently, the FOM is also computed for *k* = [*k*_0_ + *k*(*T*_i_)]/2. Besides, it is observed from Table 4 that the minimum *FOM* achieved in this study is smaller than its respective value (for the same film) from [50], which indicates that the characterization of the a-Si film presented here is more accurate than that from [50].

Besides, the data in red color from Table 1 and Table 3, and the comments from the last two paragraphs of Section 2.3, indicate that δd/$\bar{d}$ < 0.1% for either of the studied films. Furthermore, a comparison of the average film thickness from the SEM images with the computed $\bar{d}$ (shown in Figure 3b and Figure 5b), confirms that the relative error in the computation of the average film thickness does not exceed 0.1%, for both films. Moreover, these results represent achieving a relative error of 0.1% in the computation of both the average film thickness and the refractive index *n*(*λ*) of the film, as predicted in [54] and discussed in the last two paragraphs of Section 2.3.

On the other hand, it is observed from Equation (1) that *T*_sm_(*λ*) ~ *τ*_a,f_*τ*_f,s_*τ*_f,a_*x* ~ (*n*^2^ + *k*^2^)*x*, as usually *x* ≈ 1 for *λ* > min(*λ*_t_) (this can be calculated from the formula for *x*, e.g., by replacing the already known $\bar{d}$ and *k*(*λ*) for the films studied here). Respectively, *T*(*λ*) ~ *n*^2^ + *k*^2^ for *λ* > min(*λ*_t_), where *n*(*λ*) >> *k*(*λ*) for *λ* > min(*λ*_t_) (it can be observed by comparing the data for *T*(*λ*), *n*(*λ*), and *k*(*λ*), e.g., from Figure 3, Figure 4, Figure 5 and Figure 6). These two relationships indicate that the increased accuracy achieved in this paper, in the computation of *n*(*λ*), results in a significantly larger increase in the computation accuracy of the extinction coefficient *k*(*λ*) in the region *λ* > min(*λ*_t_), for each of the studied films. This fact contributes to the obtained decreased values of *FOM*, respectively, by 10% for the a-Si film compared to the data from [45] (as observed from Table 2), and by 2.8% for the a-As_98_Te_2_ film compared to the data from [47] (see Table 4).

## 5. Conclusions

Three issues are investigated for further increasing the accuracy of the characterization of a thin dielectric or semiconductor film on a substrate from *T*(*λ*) of the specimen, based on the EM.

1. Firstly, it is demonstrated that the dual transformation, based on the product *T*(*λ*)*x*_s_(λ), increases the accuracy of the envelopes *T*_+_(*λ*) and *T*_−_(*λ*) that are used in the computation of the average film thickness $\bar{d}\;$ and the film thickness non-uniformity ∆d, when the substrate is non-transparent. In practice, this approach resolves the problem of computing accurate envelopes of the interference spectrum *T*(*λ*) of a thin film on a non-transparent substrate.
2. Secondly, how to select an interval *ℓ* = [*ℓ* _1_, *ℓ* _2_] (representing the used *λ*_t_) over which the first stage of the characterization is performed most accurately, is shown. The increased accuracy of the computation of $\bar{d}\;$ and ∆d of the studied a-Si and a-As_98_Te_2_ films indicates that employing this novel concept can increase the accuracy of the characterization of every thin dielectric or semiconductor film on a substrate, based on the EM for *T*(*λ*).
3. Thirdly, the regression of *n*(*λ*) and *k*_0_(*λ*), by a polynomial of the optimized degree of 1/*λ*, is consistent with the Cauchy’s dispersion formula for materials with normal dispersion. Moreover, using only such regression eliminates the inconvenience of attempting another regression function.

The above three issues can be considered as useful supplementation to optimizing our envelope method for *T*(*λ*) (OEM for *T*(*λ*)), for thin-film characterization from [43]. In this sense, the EM implemented here, based on the algorithm from Figure 2, represents OEM for *T*(*λ*), providing and using optimized values of the characterization parameters ∆d, *ℓ* _1_ and *ℓ* _2_.

The comments from the last two paragraphs of Section 4, and our literature surveys from [43,44,45,47], indicate that the OEM characterizations presented here, corresponding to the data in red from Table 1, Table 2, Table 3 and Table 4, are undoubtedly the most accurate published optical thin-film characterizations of relatively thick films, only from *T*(*λ*).

Precise computation of both envelopes of *T*(*λ*) is needed for accurate film characterization, based on the algorithm from Figure 2, which requires the presence of at least five discernible interference extrema in *T*(*λ*). On the other hand, the number of interference extrema in *T*(*λ*) decreases with decreasing the average film thickness $\bar{d}$ (in accordance with Equation (3)). Moreover, the extinction coefficient *k*(*λ*) of dielectric or semiconductor film usually rises significantly with decreasing *λ* in the UV spectral region, which commonly leads to *x*(*λ*) → 0, *T*(*λ*) → 0 (according to Equation (1)) and the absence of interference extrema of *T*(*λ*) in the UV spectral region, for such films. The last two factors limit the applicability of the discussed OEM for *T*(*λ*), employing the algorithm from Figure 2, to dielectric or semiconductor films with average film thickness of usually at least 300 nm.

However, the reflectance spectrum *R*(*λ*) of a thin dielectric or semiconductor film, on a light-transmitting substrate, usually contains several discernible interference extrema in the UV spectral region (where there are no such extrema in the respective *T*(*λ*) of the same specimen). Correspondingly, it is expected that the OEM for *R*(*λ*) should be applicable to dielectric and semiconductor films that are significantly thinner than 300 nm. Notably, our group is in the advanced stage of development of such OEM for *R*(*λ*).

## Figures and Tables

**Table 1:** The interval *ℓ* _e_ = [*ℓ* _1e_, *ℓ* _2e_] and δd/$\bar{d}$(*ℓ*_1e_), for successively increasing *ℓ*_1e_, from the first-stage characterization of the a-Si film. The data about δd/$\bar{d}$(*ℓ*_1e_=1) (%) from this study are in blue color, and in green—from [45]; whereas the minimum value of δd/$\bar{d}$(*ℓ*_1e_) (%) is in red.

a-Si, First-Stage Characterization							from Ref. [45]
*ℓ*_e_ = [*ℓ*_1e_, *ℓ*_2e_]	[1,18]	[2,18]	[3,26]	[4,26]	[5,14]	[6,26]	[1,16]
δ$d/\bar{d}$(*ℓ* _1e_) (%)	0.143	0.146	0.165	0.168	0.0901	0.170	0.245
computed film characteristics	for *ℓ* = [5,14]$:\;\bar{d}$ = 3949.2 nm, Δd = 53.0 nm, *m*_1_ = 12						$\bar{d}$ = 3929.9 nm, Δd = 53.5 nm, *m*_1_ = 12

**Table 2:** The figure of merit *FOM* for different options about the extinction coefficient *k*(λ) of the a-Si film. The minimum of *FOM* achieved in this study is in red color, and the minimum of *FOM* from [45] is in green.

a-Si, Second-Stage Characterizations					
*FOM*	for *k* = *k*_0_	for *k* = *k*_0_ + Δ*k*	for *k*(*T*_+_)	for *k*(*T*_i_)	for [*k*_0_ + Δ*k* + *k*(*T*_i_)]/2
From ref. [45]	7.36 × 10^−3^	5.71 × 10^−3^	7.78 × 10^−3^	-	-
this study	6.65 × 10^−3^	5.19 × 10^−3^	7.41 × 10^−3^	6.91 × 10^−3^	5.80 × 10^−3^

**Table 3:** The interval *ℓ* _e_ = [*ℓ* _1e_, *ℓ* _2e_] and δd/$\bar{d}$(*ℓ*_1e_), for successively increasing *ℓ*_1e_, from the first-stage characterization of the a-As_98_Te_2_ film. The minimum of δd/$\bar{d}$(*ℓ* _1e_) (%) from this study is in red color, and the minimum of δd/$\bar{d}$(*ℓ*_1e_) (%) from [47] is in green.

a-As_98_Te_2_, First-Stage Characterization							From Ref. [47]
*ℓ*_e_ = [*ℓ*_1e_, *ℓ*_2e_]	[1,19]	[2,9]	[3,9]	[4,9]	[5,9]	[6,9]	[2,12]
δ$d/\bar{d}$(*ℓ* _1e_) (%)	0.308	0.0857	0.0426	0.0455	0.0491	0.0497	0.133
computed film characteristics	for *ℓ* = [3,9]: $\bar{d}$ = 1983.2 nm, Δd = 23.9 nm, *m*_1_ = 4.5						$\bar{d}$ = 1983.8 nm, Δd = 22.7 nm, *m*_1_ = 4.5

**Table 4:** The figure of merit *FOM* for different options about the extinction coefficient *k*(λ) of the a-As_98_Te_2_ film. The minimum of *FOM* achieved in this study is in red color, and the minimum of *FOM* from [47] is in green.

a-As_98_Te_2_, Second-Stage Characterizations					
*FOM*	for *k* = *k*_0_	for *k* = *k*_0_ + Δ*k*	for *k*(*T*_+_)	for *k*(*T*_i_)	for [*k*_0_ + *k*(*T*_i_)]/2
from [47]	4.36 × 10^−3^	4.26 × 10^−3^	3.96 × 10^−3^	3.74 × 10^−3^	-
this study	3.89 × 10^−3^	3.89 × 10^−3^	4.38 × 10^−3^	3.87 × 10^−3^	3.64 × 10^−3^

## Data Availability

The data underlying this article will be shared on reasonable request from the corresponding author.
